# CD8^+^ T Cells Restrict *Yersinia pseudotuberculosis* Infection: Bypass of Anti-Phagocytosis by Targeting Antigen-Presenting Cells

**DOI:** 10.1371/journal.ppat.1000573

**Published:** 2009-09-04

**Authors:** Molly A. Bergman, Wendy P. Loomis, Joan Mecsas, Michael N. Starnbach, Ralph R. Isberg

**Affiliations:** 1 Department of Molecular Biology and Microbiology, Tufts University School of Medicine, Boston, Massachusetts, United States of America; 2 Department of Microbiology and Molecular Genetics, Harvard Medical School, Boston, Massachusetts, United States of America; 3 Howard Hughes Medical Institute, Tufts University School of Medicine, Boston, Massachusetts, United States of America; The Rockefeller University, United States of America

## Abstract

All *Yersinia* species target and bind to phagocytic cells, but uptake and destruction of bacteria are prevented by injection of anti-phagocytic Yop proteins into the host cell. Here we provide evidence that CD8^+^ T cells, which canonically eliminate intracellular pathogens, are important for restricting *Yersinia*, even though bacteria are primarily found in an extracellular locale during the course of disease. In a model of infection with attenuated *Y. pseudotuberculosis*, mice deficient for CD8^+^ T cells were more susceptible to infection than immunocompetent mice. Although exposure to attenuated *Y. pseudotuberculosis* generated T_H_1-type antibody responses and conferred protection against challenge with fully virulent bacteria, depletion of CD8^+^ T cells during challenge severely compromised protective immunity. Strikingly, mice lacking the T cell effector molecule perforin also succumbed to *Y. pseudotuberculosis* infection. Given that the function of perforin is to kill antigen-presenting cells, we reasoned that cell death marks bacteria-associated host cells for internalization by neighboring phagocytes, thus allowing ingestion and clearance of the attached bacteria. Supportive of this model, cytolytic T cell killing of *Y. pseudotuberculosis*–associated host cells results in engulfment by neighboring phagocytes of both bacteria and target cells, bypassing anti-phagocytosis. Our findings are consistent with a novel function for cell-mediated immune responses protecting against extracellular pathogens like *Yersinia*: perforin and CD8^+^ T cells are critical for hosts to overcome the anti-phagocytic action of Yops.

## Author Summary

Pathogenic *Yersinia* are bacteria that cause diverse diseases such as gastroenteritis and plague. *Yersinia* binds to specialized immune cells called macrophages, which attempt to engulf and destroy the bacteria. The bacteria resist destruction by injecting proteins called Yops into macrophages, which stops the engulfment process. *Yersinia* thus survives as attached but extracellular bacteria to cause disease. *Yersinia* disease can be prevented by immunization. In this study, we identified one mechanism of protective immunity—that host cells called CD8^+^ T lymphocytes are important to restrict *Yersinia* infection. This observation is unusual because CD8^+^ T cells generally protect against intracellular pathogens: T cells destroy the host cell harboring the pathogen, thus preventing the pathogen's replication. We present data consistent with the model that CD8^+^ T cells can also restrict extracellular bacteria by showing that T cells target host cells with extracellularly attached *Yersinia*, thus allowing the host cells and associated bacteria to be engulfed and removed by neighboring macrophages.

## Introduction

Three *Yersinia* species cause disease in humans: *Y. pestis*, *Y. pseudotuberculosis*, and *Y. enterocolitica* [1]. Plague results from *Y. pestis*, introduced via fleabite or contaminated aerosols, and manifests in bubonic, septicemia, and pneumonic forms [2]. Oral ingestion of *Y. pseudotuberculosis* or *Y. enterocolitica* from contaminated food or water causes yersiniosis, which is typified by gastroenteritis and mesenteric lymphadenitis, with occasional arthritic sequelae [3]. Despite differing infection routes and disease manifestations, all *Yersinia* species demonstrate a tropism for lymphoid tissue and an ability to disseminate from initial infection sites to colonize systemic organ sites, where unchecked bacterial replication causes fatal disease [4],[5].

*Yersinia* express a battery of virulence factors to cause disease in susceptible human and animal hosts. Enteric *Yersinia* express several adhesins in order to bind to host cells and penetrate the intestinal epithelium [6]–[12]. *Y. pestis* expresses a different subset of adhesins to mediate host cell binding and facilitate dissemination [13]–[16]. Adhesion of *Yersinia* to host cells is required for delivery of *Yersinia* outer proteins (Yops) into the host cell cytoplasm via a specialized secretion machine called the type III secretion system (T3SS) [17]. *Yersinia* express six Yops; YopE, H, J (also called P), M, O, and T [18], which perturb eukaryotic cell signaling pathways, resulting in inhibition of phagocytosis, alteration of cytokine production [18], and direct intoxication of phagocytes during animal infections [19]. The importance of Yop-induced inhibition of phagocytosis is supported by two lines of evidence: 1) Yop-deficient *Yersinia* species fail to cause disease [20]–[23], and 2) histological studies demonstrate that *Yersinia* localize to the extracellular space of infected tissues [24]–[26]. This anti-phagocytosis phenotype can be easily reproduced during *Yersinia* association with tissue culture cells [27]. Delivery of Yops requires intimate attachment, as bacteria lacking defined adhesion factors are defective for Yop translocation [28]. Thus, the combined action of adhesins and Yops synergize to localize *Yersinia* in a discrete niche as extracellularly attached bacteria [29].

T cells survey for pathogens by virtue of T cell receptor recognition of antigen bound to major histocompatibility complex (MHC) on the surface of infected host cells. CD4^+^ T cells recognize exogenous antigen from vacuolar pathogens that is presented by MHC II. CD8^+^ T cells respond to cytoplasmic antigens, with processed peptide complexed with MHC I [30]. A requirement for cell-mediated immune responses against *Yersinia* has been dismissed, given that *Yersinia* is an extracellular pathogen and that immune serum is sufficient to protect mice against virulent challenge [31],[32]. The observed intracellular localization of Yop proteins [33], however, has suggested that T cells may respond to Yop-derived antigens. Moreover, there is evidence showing that CD4^+^ and CD8^+^ T cells are required for protection against *Y. pestis* and *Y. enterocolitica* in animal models of infection [34]–[36]. The mechanism of T cell restriction of *Yersinia* is unknown. Studies have suggested that IFNγ secretion by T cells is important [37], presumably by activating antimicrobial functions in phagocytes [38]. This suggestion explains the function of *Yersinia*-specific CD4^+^ T cells, but may only partially account for the mechanism of CD8^+^ T cell restriction of the bacteria. CD8^+^, or cytolytic T cells (CTLs), can eliminate pathogen-harboring cells by inducing death of target cells, which removes a niche for intracellular pathogen replication [39]. The possibility that CTL-induced host cell death could interfere with disease by extracellular pathogens has not been addressed.

We hypothesized that the intimate association of *Y. pseudotuberculosis* with host cells may allow recognition by CD8^+^ T cells and result in restriction of disease. To test this hypothesis, a model of murine infection with live attenuated *Y. pseudotuberculosis* was used to determine the disease susceptibility of mice defective in specific arms of cell-mediated immunity. CD8^+^ T cells and perforin-dependent cytolysis were critical for protecting against *Y. pseudotuberculosis*. CD8^+^ T cell recognition of target cells bypasses anti-phagocytosis by *Y. pseudotuberculosis*, which is consistent with the model that restriction results from CTLs marking host cells and attached bacteria for phagocytic removal.

## Results

### Attenuated *Y. pseudotuberculosis* Colonization of Mice Induces Protective Immunity

**Figure 1:**
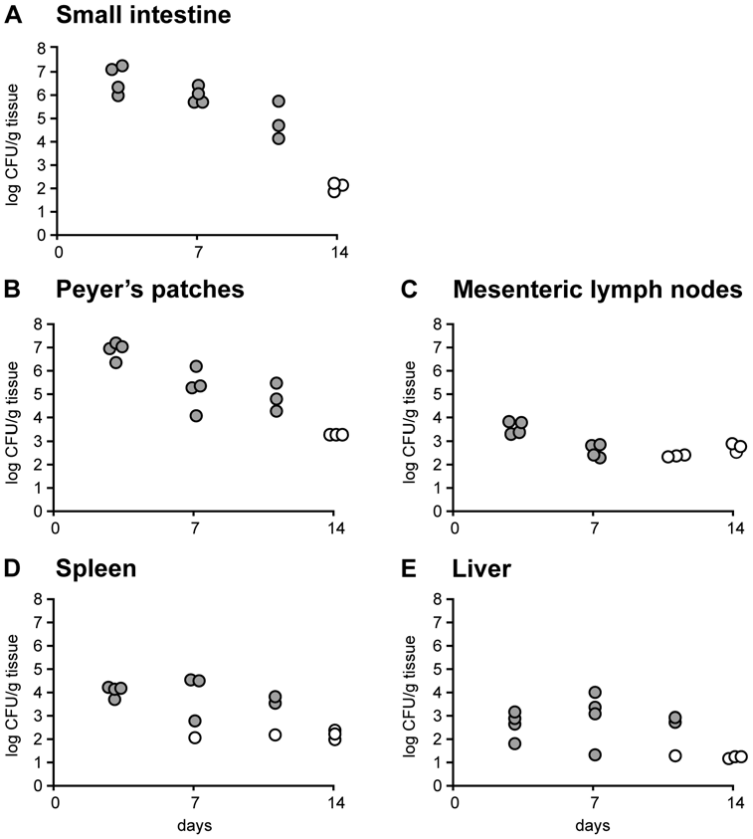
*Y. pseudotuberculosis ksgA^−^* is Attenuated in a Murine Model of Infection. C57BL/6 mice were orally inoculated with 5×10^8^ CFU of mutant *ksgA^−^* strain and mice sacrificed at days 3, 7, 11 and 14 post-inoculation. The number of bacteria in the small intestine (A), Peyer's patches (B), mesenteric lymph nodes (C), spleen (D) and liver (E) was determined by plate assay and normalized to gram tissue weight. Open symbols indicate that the bacterial numbers were below the limit of detection. Each symbol indicates one mouse.

As naïve mice rapidly succumb to disease caused by virulent *Y. pseudotuberculosis* [4],[5], we instead used the attenuated *ksgA^−^* strain [40] to investigate how CD8^+^ T cells protect against *Y. pseudotuberculosis*. The absence of KsgA results in a lowered replication rate in culture relative to wild-type bacteria [41] due to the loss of dimethylation of 16S rRNA [42]. To confirm that *Y. pseudotuberculosis ksgA* ^−^ attenuates virulence in animal hosts, C57BL/6 mice were orally inoculated with 5×10^8^ colony-forming units (CFU) of the *ksgA^−^* strain and bacterial burden was followed in target organs over time. Mice inoculated with the mutant showed detectable levels of bacteria in all organs examined for the first ten days post-inoculation, after which the levels dropped below the limit of detection (**Figure 1**), and most animals survived (data not shown). The small intestine and Peyer's patches were colonized at higher levels than the mesenteric lymph nodes, spleen or liver. In contrast, C57BL/6 mice orally inoculated at the same dose with the virulent parental strain supported increasingly greater levels of bacteria until eventually succumbing to disease eight days post-inoculation [43]. The *ksgA^−^* strain was attenuated following a non-intestinal route of inoculation, as well. After intravenous delivery, *ksgA^−^* bacteria colonized the spleen and liver at lower levels than the parental strain at day 5 post-inoculation and at levels approaching a known avirulent strain lacking the ability to translocate Yops into cells (*yopB^−^* [44]) (**Figure S1**).

Prior exposure to attenuated pathogens can generate protective immunity against subsequent challenge with fully virulent organisms [45]–[47]. Previous oral inoculation with *Y. pseudotuberculosis ksgA^−^* bacteria conferred protection against the virulent YPIII pIB1 comparable to a published vaccine strain that overproduces Dam methylase [48] (91% versus 100% survival, **Table 1**), whereas all naïve mice succumbed to challenge (0% survival). Moreover, orally immunized mice were protected against challenge with intravenously administered virulent bacteria (100%), indicating that the specificity of the response was not restricted to antigens expressed by bacteria at the initial immunization site. Immunization with *ksgA* ^−^ bacteria, which is derived from the YPIII pIB1 background [40], also protected against oral challenge with strain IP2666, another virulent *Y. pseudotuberculosis* strain [49] capable of causing fatal disease in naïve mice (data not shown). These results demonstrate that exposure to attenuated *Y. pseudotuberculosis ksgA^−^* bacteria generates immune responses sufficient to protect against virulent disease.

**Table 1:** Immunization with the *ksgA* ^−^ Mutant Protects Against Challenge by Virulent *Y. pseudotuberculosis*.

Immune Status^a^	% Survival (N/N)^c^
naïve	0 (0/9)
Dam^OP^	100 (4/4)
*ksgA^−^*	91 (11/12)
*ksgA^−^* b	100 (4/4)

a Mice were orally immunized with the indicated strain and 60–90 days post-immunization were orally challenged with 2×10^9^ CFU virulent *Y. pseudotuberculosis* strain YPIII pIB1.

b Immune mice were intravenously challenged with 5×10^2^ virulent *Y. pseudotuberculosis*.

c Failure to survive was scored if mice succumbed to challenge or were moribund.

### Attenuated *Y. pseudotuberculosis* Infection Induces Humoral and Cell-Mediated Immune Responses

**Figure 2:**
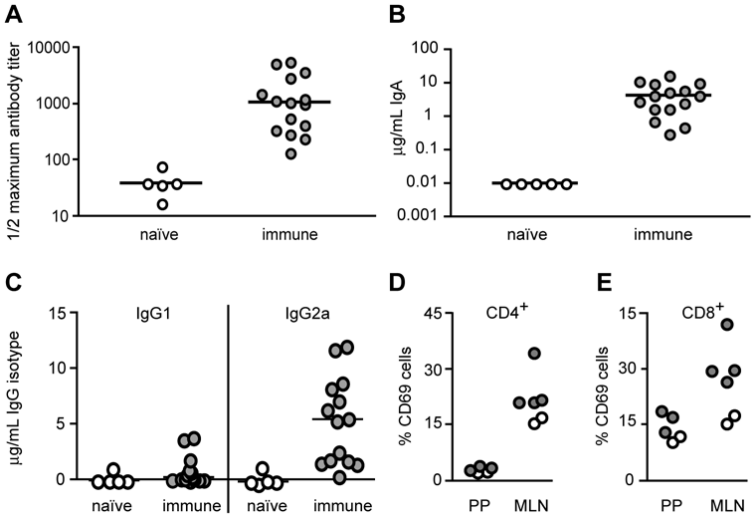
Colonization by *ksgA^−^* Induces T_H_1-type Antibodies and Activates T Cell Responses. Naïve mice or mice orally inoculated with *ksgA^−^* bacteria 60 days prior were sacrificed, sera harvested and assayed for *Y. pseudotuberculosis*-specific antibodies. Shown are the half-maximal titer of “total” serum antibodies (combined IgM, IgA, IgD, IgG) (A), and the concentrations of IgA (B), and IgG1 and IgG2a serum antibodies (C) specific to total *Yersinia* antigen. 7 days post-oral inoculation, the percentage of CD69^+^ CD4^+^ (D) or CD69^+^ CD8^+^ (E) T cells present in the Peyer's patches (PP) and mesenteric lymph nodes (MLN) was determined. Each symbol represents one animal: filled symbols are animals exposed to *Y. pseudotuberculosis* and open symbols are naïve animals.

Correlates of protective immunity include the presence of antigen-specific antibodies and T cell responses following antigenic exposure. The total antibody response to *Y. pseudotuberculosis* was significantly higher in sera from *ksgA^−^*-immunized mice than from naïve mice, with a 28 fold greater median value for the half-maximal antibody titer in immune animals (**Figure 2A**, *P* = 0.0012). Antibody isotyping revealed that *ksgA^−^*-immune mice possessed anti-*Yersinia* IgA antibodies, as would be predicted from a mucosal immunization regimen (**Figure 2B**), as well as robust IgG2a and detectable IgG1 responses (data not shown). *Y. pseudotuberculosis*-immune mice displayed a significantly more IgG2a than IgG1 isotypic antibodies (**Figure 2C**, median ratios 5.9 and 0.4, respectively, *P* = 0.0004), indicative of a T_H_1-type T cell response [50]. Correspondingly, CD4^+^ and CD8^+^ T cells were activated at day seven post-inoculation with the *ksgA* ^−^ mutant, as determined by an increase in CD69^+^ lymphocytes in mesenteric lymph nodes as compared to naïve animals, although activated lymphocytes were not detected in the *ksgA^−^*-colonized Peyer's patches at this time point (**Figure 2D, E**). The increase in the percentage of activated lymphocytes was due to an increase in the number of activated cells relative to the total number of lymphocytes. The number of CD8^+^ lymphocytes was similar between animals regardless of the presence of bacteria (data not shown), but the number of activated CD8^+^ lymphocytes was higher in animals harboring bacteria. Taken together, these results indicate that both humoral and cell-mediated immune responses are stimulated in mice exposed to attenuated *Y. pseudotuberculosis*.

### CD8^+^ T Cells Are Required to Protect Against *Y. pseudotuberculosis* Infection

**Figure 3:**
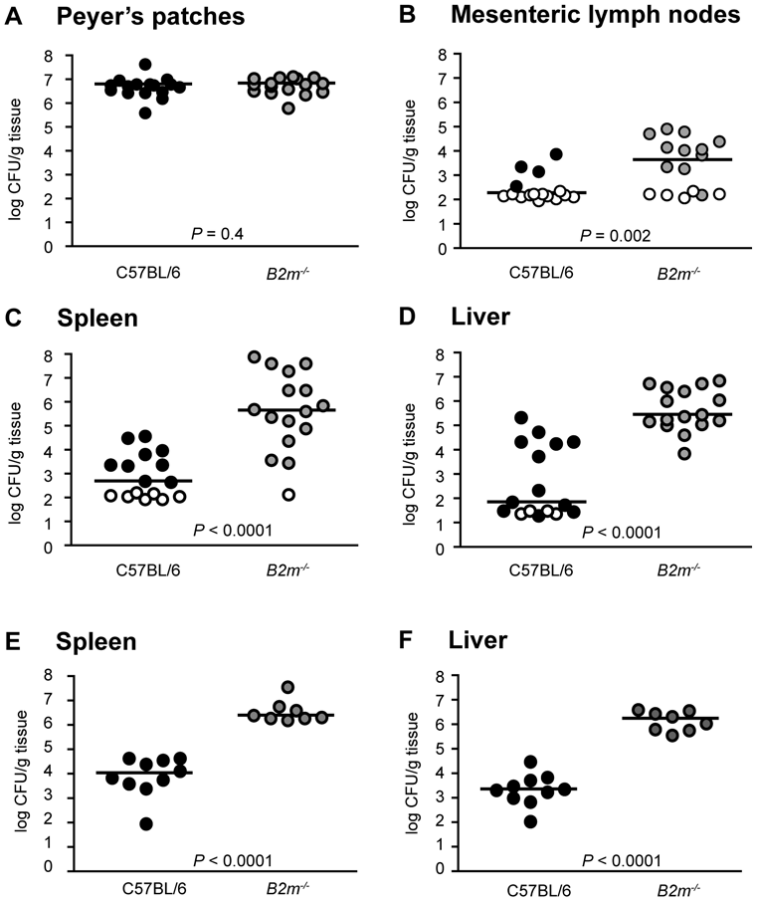
β2-Microglobulin-Deficient Mice Are Highly Susceptible to Attenuated *Y. pseudotuberculosis*. C57BL/6 (black circles) or *B2m* ^−/−^ (grey circles) mice were orally inoculated with 5×10^8^ CFU attenuated *ksgA^−^* bacteria, sacrificed at day 8–10 post-inoculation and bacterial burden assayed in Peyer's patches (A), mesenteric lymph nodes (B), spleen (C), and liver (D). Open symbols indicate values below the limit of detection for that organ. Alternatively, C57BL/6 mice or *B2m^−/−^* mice were intravenously inoculated with 2×10^2^ CFU *ksgA^−^* bacteria, sacrificed at day 10 post-inoculation and bacteria burden in the spleen (E) and liver (F) determined. Each symbol represents one animal, lines are median values. Data shown is pooled from two-three separate experiments.

The observation that exposure to attenuated *Y. pseudotuberculosis* activates CD8^+^ T cells was intriguing, given that CD8^+^ T cells are thought to target intracellular pathogens, while *Yersinia* species are generally regarded to be extracellular pathogens. To investigate if CD8*^+^* T cells restrict *Y. pseudotuberculosis* colonization, mice deficient for β2-microglobulin (*B2m^−/−^*), which lack MHC Class I and thus lack CD8^+^ T cells [51],[52], were inoculated with the *ksgA^−^* mutant. At eight days post-inoculation, the *ksgA^−^* mutant colonized the Peyer's patches (**Figure 3A**) and small intestine (data not shown) of C57BL/6 and *B2m^−/−^* mice at similar levels, indicating that *Y. pseudotuberculosis* replicates in the gastrointestinal tract independently of β2-microglobulin-dependent mechanisms. However, the mesenteric lymph nodes, spleen and liver of *B2m^−/−^* mice were colonized at significantly higher levels with attenuated bacteria as compared to C57BL/6 mice, demonstrating that *B2m^−/−^* mice are highly susceptible to the *ksgA* ^−^ mutant (**Figure 3B, C, D**). In addition to lacking cell-mediated immune functions, *B2m^−/−^* mice have an additional phenotype of iron overload, similar to human hemochromatosis [53]. Given that hemochromatosis is a risk factor for extraintestinal infection by enteric *Yersinia* in humans [54], we evaluated the colonization of *Y. pseudotuberculosis* in *B2m^−/−^* mice following parenteral inoculation. This allows the infection route to bypass the intestine and avoid hemochromatosis-dependent enhancement of extraintestinal dissemination. At nine to ten days post-inoculation, intravenously delivered *ksgA^−^* mutant bacteria colonized the spleen and liver of *B2m^−/−^* knockout animals at significantly higher levels than observed in C57BL/6 mice (**Figure 3E, F**), indicating that the enhanced susceptibility of the knockout mice to Y. *pseudotuberculosis* cannot entirely be attributed to increased extraintestinal dissemination. Therefore CD8^+^ T cells, or cells and/or functions requiring β2-microglobulin, are necessary to eliminate attenuated *Y. pseudotuberculosis*.

**Figure 4:**
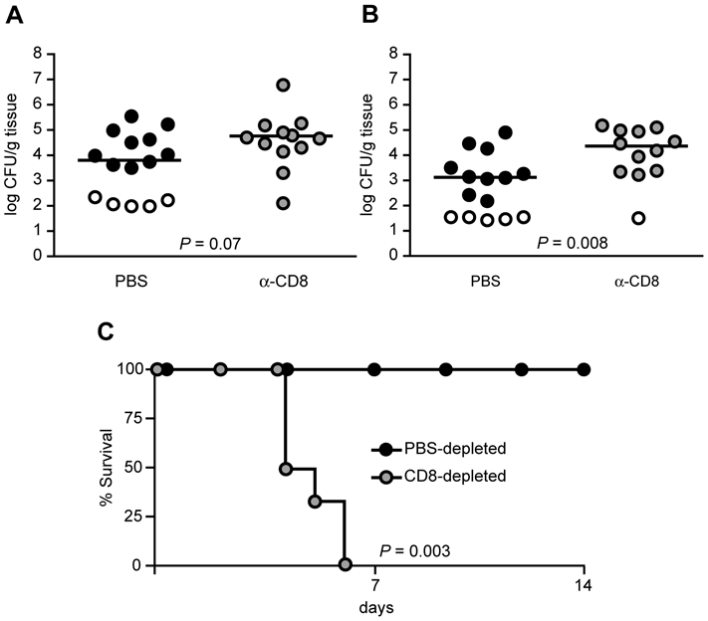
CD8^+^ T cells are Required to Protect Against *Y. pseudotuberculosis*. Naïve mice were given phosphate-buffered saline (PBS, black circles) or CD8-depleting antibody 2.43 (α-CD8, grey circles) prior and subsequent to intravenous inoculation with 10^2^ CFU *ksgA^−^* bacteria, sacrificed at day 14 post-inoculation and CFU enumerated in the spleen (A) and liver (B). Open symbols indicate the limit of detection for that organ, each symbol represents one mouse, and lines are median values. Data shown is pooled from three separate experiments. (C) To examine protection in immune animals, at 60 days post-oral *ksgA^−^* immunization, mice received PBS (black circles) or CD8-depleting antibody 2.43 (α-CD8, grey circles) prior and subsequent to intravenous inoculation with 10^4^ CFU virulent YPIII pIB1 (*ksgA^+^*) bacteria. Morbidity and mortality was followed for 14 days post-challenge and % survival shown by Kaplan-Meier plots. Data is representative of two experiments.

To more specifically isolate the role of CD8^+^ T cells in controlling colonization with attenuated *Y. pseudotuberculosis*, C57BL/6 mice were depleted of CD8^+^ T cells [55] (Materials and Methods) and inoculated with *ksgA^−^* bacteria. Bacteria were delivered directly to the systemic organs by intravenous injection, to ensure that the mutant was introduced into organ sites that have a high likelihood of depletion. This was effective in reducing the scatter found in CFU isolated from systemic tissue sites after oral inoculation, presumably resulting from bottlenecking associated with extraintestinal dissemination [43]. At 14 days post-inoculation, mice depleted of CD8^+^ T cells displayed a 1.2 and 1.4 log increase in CFU in the spleen and liver, respectively (**Figure 4A, B**), as compared to mock-depleted mice, although differences were only statistically significant in the liver (*P* = 0.008). More striking was the frequency of colonization: while some of the mock-depleted animals showed no colonization (33%, 5 of 15 mice), all of the CD8-depleted mice contained *ksgA* ^−^ bacteria in either the liver or spleen (100%, 12 of 12 mice). *CD8* ^−/−^ mice [56] also demonstrated increased susceptibility to systemic colonization with *ksgA^−^* bacteria at day 14 post-inoculation (median value of 10^4^ versus 10^2^ CFU/gram tissue in livers of *CD8* ^−/−^ as compared to C57BL/6 mice, *P* = 0.02).

Although these experiments addressed the requirement for CD8^+^ T cells during colonization with attenuated *Y. pseudotuberculosis* in naïve animals, that is, those not previously exposed to the bacterium, the observed phenotype of increased colonization in CD8-deficient mice was detected only at 14 days post-inoculation, at a time when adaptive immune mechanism are likely involved in protection. To demonstrate that the requirement for CD8^+^ T cells is associated with enhanced adaptive immunity to *Y. pseudotuberculosis*, and to show that CD8^+^ T cell responses are effective against fully virulent bacteria, mice orally immunized with the *ksgA^−^* mutant were depleted of CD8^+^ T cells prior to and following intravenous challenge with wild-type *Y. pseudotuberculosis*. 100% of depleted mice succumbed to virulent bacteria within one week post-inoculation, whereas all mock-depleted immune mice survived challenge (**Figure 4C**); the two survival curves were significantly different (*P* = 0.003). These results demonstrate that effective protective immunity to *Y. pseudotuberculosis* requires the presence of CD8^+^ T cells.

### Perforin Function Is Critical to Limit *Y. pseudotuberculosis* Infection

**Figure 5:**
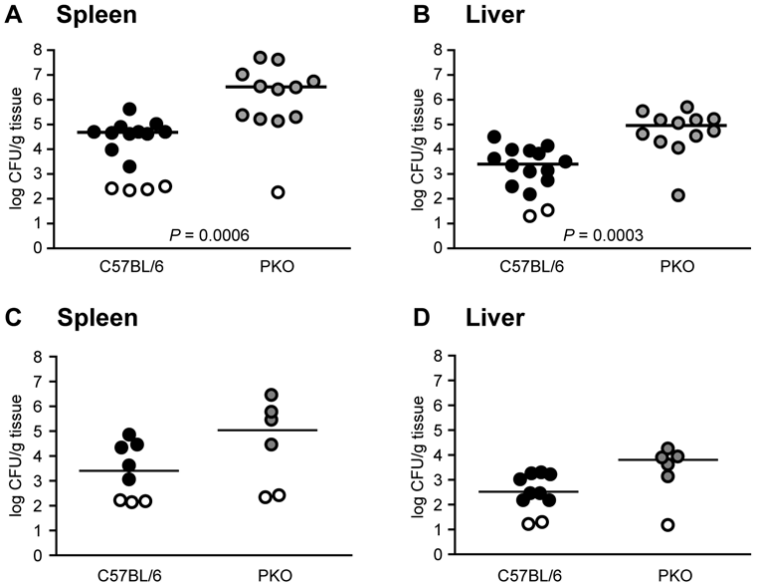
Perforin Is Required to Restrict *Y. pseudotuberculosis* Colonization. C57BL/6 (black circles) or perforin-deficient animals (*PKO*, grey circles) mice were intravenously inoculated with 10^2^ attenuated *ksgA^−^* bacteria and mice sacrificed at day 14–15 post-inoculation to assay bacterial burden in spleen (A), and liver (B). To examine protection in immune animals, at 60 days post-intravenous *ksgA^−^* immunization, mice were intravenously challenged with 10^3^ CFU virulent YPIII pIB1 (*ksgA^+^*) bacteria and bacterial burden assayed in the spleen (C) and liver (D) at day 7 post-challenge. Open symbols indicate the limit of detection for that organ. Each symbol represents one mouse, lines are median values. Data shown is pooled from two-three separate experiments.

CD8^+^ T cells mediate protective immune responses via secreting cytokines or by directly killing target cells and removing a niche for pathogen intracellular replication. Cytolytic T cells (CTLs) can kill target cells via localized secretion of perforin, which inserts into target cell membranes and facilitates entry of other secreted molecules involved in target cell killing such as granzymes and granulysins, interrupting growth of intracellular pathogens in target cells [57]. To address the requirement for perforin in protecting against *Y. pseudotuberculosis* replication, perforin-deficient (PKO) [58] and -sufficient mice were intravenously inoculated with the *ksgA^−^* mutant and bacterial colonization in systemic organ sites was examined. *PKO* mice were highly susceptible to growth of attenuated *Y. pseudotuberculosis*, with increased bacterial burden as compared to C57BL/6 mice in both spleen (1.4 log CFU median increase, *P* = 0.0006) and liver (1.5 log CFU median increase, *P* = 0.0003) at day 14–15 post-inoculation (**Figure 5A, B**). These results demonstrate that perforin function is required to eliminate attenuated *Y. pseudotuberculosis*, and suggest that CD8^+^ T cells limit bacterial replication by perforin-dependent mechanisms. To determine if perforin plays a role in protective immune responses to *Y. pseudotuberculosis*, we repeated immunizations of perforin-deficient animals and C57BL/6 mice, extending the time post-inoculation beyond 14 days. Perforin deficiency impaired the immune response even to the attenuated strain, as 3 of the 9 knockout animals succumbed to immunizing strain by 3 weeks post-inoculation, as compared to 1 of 10 control animals. Those animals surviving *ksgA^−^* immunization were then subjected to challenge with virulent *Y.* pseudotuberculosis. Mice intravenously immunized with 10^2^ CFU *ksgA* ^−^ 60 days prior were challenged via the same route with 10^3^ CFU YPIII pIB1, a dose that is virulent for naïve C57BL/6 animals (data not shown). At seven days post-challenge, perforin-deficient *Y. pseudotuberculosis*-immune mice demonstrated higher bacterial burden in spleens and livers than control immune C57BL/6 mice (**Figure 5C, D**); knockout animals with detectable colonization had significantly higher median CFU values: in the spleen, 5.67 versus 4.39 for perforin-deficient versus C57BL/6 (p-value = 0.02); in the liver, 3.93 versus 3.08 for perforin-deficient versus C57BL/6 mice, p-value = 0.01). Thus, perforin-deficient animals immunized with *Y. pseudotuberculosis* were more susceptible to challenge with fully virulent bacteria than control C57BL/6 mice. The increased susceptibility of perforin-deficient mice both to primary infection with attenuated *Y. pseudotuberculosis* and challenge infection with virulent bacteria indicates that perforin is required during the initial and memory phases of the immune response. During primary exposure to attenuated *Y. pseudotuberculosis*, the enhanced susceptibility in perforin-deficient mice as compared to mice lacking CD8^+^ T cells could result from the absence of perforin in other cell types such as natural killer (NK) and NK T cells [39]. Even so, there is no established model for how any of these cells could clear extracellular pathogens in a perforin-dependent fashion.

### *Y. pseudotuberculosis* Maintains an Extracellular Locale in the Absence of CD8^+^ T cells or Perforin

Cell-mediated immune responses are thought to eliminate intracellular pathogens. While members of the *Yersinia* genus are generally considered to be extracellular pathogens, *Y. pestis* and *Y. pseudotuberculosis* have been shown to replicate inside cultured macrophages if the bacteria express low levels of Yops [59],[60]. This raises the possibility that a reservoir of intracellular bacteria may seed the extracellular bacterial population, allowing *Yersinia*-specific CTLs to restrict *Yersinia* colonization by targeting phagocytes with intracellular bacteria. This model predicts that mice with impaired CTL function have a larger fraction of intracellular *Yersinia* than do wild type mice. To test this hypothesis, we determined the levels of intracellular *Y. pseudotuberculosis* in mice lacking perforin, β2-microglobulin, or CD8^+^ T cells, using a previously described “*ex vivo*” gentamicin protection assay ([61] see Materials and Methods).

**Figure 6:**
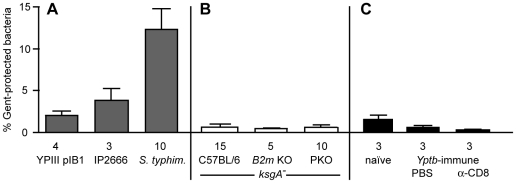
*Y. pseudotuberculosis* Localizes Extracellularly in the Spleen Despite the Absence of Cytolytic T cell Control. Single cell suspensions of spleens from animals exposed to bacteria were assayed for the presence of intracellular bacteria using a gentamicin protection assay – spleen cells were assayed for CFU without and with gentamicin treatment, and % gentamicin-protected bacteria calculated (see Materials and Methods). (A) C57BL/6 mice were intravenously inoculated with *Y. pseudotuberculosis* strain YPIII pIB1 or IP26666 (2×10^3^ CFU), or *S. typhimurium* strain SL1344 (5×10^2^ CFU) and sacrificed 3–5 days post-inoculation. (B) C57BL/6, PKO and *B2m^−/−^* mice were intravenously inoculated with 10^2^ CFU *ksgA^−^* bacteria and sacrificed after 10–14 days post-inoculation. (C) Naïve or *Y. pseudotuberculosis*-immune C57BL/6 mice treated with PBS or CD8-depleting antibody 2.43 (see Figure 4 legend) were challenged with 10^4^ virulent YPIII pIB1 and sacrificed day 4 post-challenge. Values show means and S.E.M. for several experiments; number of animals used for each condition is listed below each experimental condition.

Mice were intravenously inoculated with *Y. pseudotuberculosis*, and at the indicated time points post-inoculation, spleen cell suspensions were treated with gentamicin (**Figure 6**). As a positive control for intracellular bacteria, animals were also inoculated with *S. typhimurium* [61]. Spleen cell suspensions from *Y. pseudotuberculosis*-inoculated C57BL/6 harbored lower numbers of gentamicin-protected bacteria at 3–4 days post-inoculation as compared to animals inoculated with *S. typhimurium* 5 days prior (**Figure 6A**). The *phoP^−^* YPIII pIB1 strain displayed reduced numbers of intracellular bacteria in spleen suspensions (**Figure 6A**) and in cultured macrophages (**Figure S2**) as compared to a *phoP* ^+^ strain (IP2666), confirming prior observations of the importance of PhoP for intracellular survival replication [62],[63].

Using the gentamicin protection assay, we then examined the yields of intracellular bacteria in PKO, *B2m^−/−^* and control C57BL/6 mice at 10–14 days post-inoculation with the YPIII *ksgA^−^* strain, when bacterial burdens were highest in the spleen (**Figures 3** **,** **5**). The *ksgA* ^−^ mutant displayed very low yields of gentamicin protection in spleen cell suspensions from C57BL/6 mice (**Figure 6B**). The protection efficiencies of bacteria isolated from spleens of animals lacking either β2-microglobulin or perforin were similar to those obtained from control C57BL/6 cell suspensions (**Figure 6B**). Moreover, when *ksgA^−^* immunized mice were depleted of CD8^+^ T cells during challenge with wild type *Y. pseudotuberculosis*, the fraction of gentamicin-protected bacteria in spleen cells was no higher than in mock-depleted mice or age-matched naïve mice at day 4 post-challenge (**Figure 6C**). These observations indicate that mice lacking β2 microglobulin, perforin, or CD8^+^ T cells do not show increased susceptibility to *Y. pseudotuberculosis* as a result of an increased burden of intracellular bacteria, but are consistent with a mechanism of cell-mediated immune restriction against *Y. pseudotuberculosis* that functions to eliminate extracellular bacteria.

### CTLs Target *Y. pseudotuberculosis*-Associated Antigen-Presenting Cells But Do Not Alter Bacterial Localization

**Figure 7:**
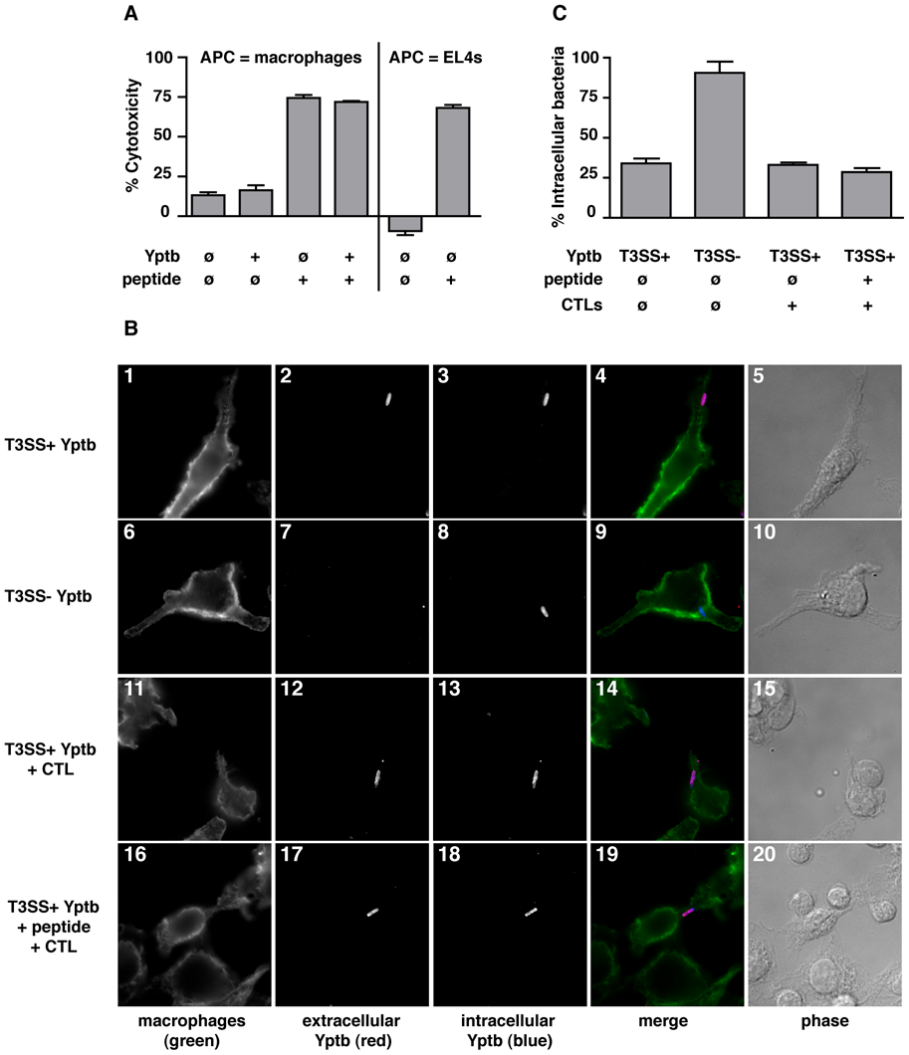
CTL Targeting of *Y. pseudotuberculosis*-Associated APCs Does Not Induce Bacterial Uptake by APCs. (A) Bone-marrow derived macrophages were pulsed with CrpA_63–71_ peptide, infected with *Y. pseudotuberculosis* and exposed to CrpA_63–71_-specific CTLs at a ratio of 10∶1 CTL∶APC for four hours, after which culture supernatants were evaluated for the presence of the cytoplasmic enzyme lactate dehydrogenase (LDH). The EL4 thymoma cells were used as control antigen-presenting cells. Released LDH was normalized to the maximal amount of released LDH and % cytotoxicity calculated (see Methods). Shown are the mean and standard error of the mean (SEM) for triplicate samples. (B) Macrophages were treated with peptide and *Y. pseudotuberculosis*, exposed to CTLs at a ratio of 10∶1 CTL∶APC for 15 minutes, then cells were fixed and assayed for the presence of extra- and intracellular bacteria by immunofluorescence microscopy. Representative images from one of four experiments are shown. Macrophage treatment conditions: panels 1–5 T3SS^+^ bacteria, panels 6–10 T3SS^−^ bacteria, panels 11–15 T3SS^+^ bacteria and CrpA-specific CTLs (no peptide), panels 16–20 T3SS^+^ bacteria, CrpA_63–71_ peptide, and CrpA-specific CTLs. Panels from left to right show CD11b^+^ macrophages (green), extracellular *Yersinia* (red), internalized *Yersinia* (blue), merged images, and phase contrast. White bar represents 10 µm. (C) Quantification of intracellular bacteria: infected macrophages were scored for intra- and extracellular bacteria and % intracellular *Y. pseudotuberculosis* calculated. Values shown are mean and SEM for triplicate samples, and results are representative of three experiments.

We next considered the possibility that the predominant locale for *Yersinia*, extracellular but attached to host cells, allows cytolytic CD8^+^ T cells (CTLs) to directly inhibit bacterial replication, by instructing antigen-presenting cells (APCs) to internalize or kill surface-bound *Y. pseudotuberculosis*. To test this possibility, we established a cell culture system that recapitulates CD8^+^ T cell cytolysis of APCs harboring *Y. pseudotuberculosis*. We used a surrogate CTL antigen, as natural bacterial antigens recognized by CD8^+^ T cells from *Yersinia*-immune mice have not been identified. Primary macrophages, a target of *Yersinia in vivo* [19], were used as APCs and incubated with CrpA_63–71_ peptide, which is recognized by CTLs derived from *Chlamydia*-immune mice [64]. Peptide-presenting macrophages were effectively lysed by a CrpA_63–71_-specific CTL line, regardless of the presence of bacteria (**Figure 7A**), indicating that *Y. pseudotuberculosis* did not impair CTL killing of APCs. In contrast, bacteria-associated macrophages lacking peptide showed high viability following T cell exposure, similar to the macrophages exposed to media only. The degree of CTL-induced macrophage death was comparable to that observed with a standard antigen-presenting cell line (thymic carcinoma cell line EL4, **Figure 7A**), confirming that macrophages are targeted equally as well as conventional APCs. These results demonstrate that antigen-specific CTLs kill cultured APCs harboring surface-attached *Y. pseudotuberculosis*.

We then examined the consequence of CD8^+^ T cell targeting upon *Y. pseudotuberculosis* internalization by the target host cell. APCs were incubated with bacteria, pulsed with peptide and exposed to CTLs, and bacterial internalization by the APCs was assessed using an immunofluorescence protection assay on fixed cells (see Methods). Control incubations of APCs with wild-type *Y. pseudotuberculosis* or mutant bacteria deficient for translocation of anti-phagocytosis proteins (T3SS^+^ or T3SS^−^, respectively) demonstrated that wild-type bacteria localize as extracellularly-attached bacteria (**Figure 7B**, panels 1–5, pink bacteria), while T3SS^−^ bacteria were found intracellularly (**Figure 7B**, panels 6–10, blue bacteria), as has been previously observed [65]. CTL exposure to APCs with associated *Y. pseudotuberculosis* failed to alter the localization of wild-type bacteria, regardless of whether peptide was added (**Figure 7B**, panels 11–15, no peptide; 16–20, plus peptide, **Figure 7C**). Altering the CTL exposure time or the CTL-to-APC ratio did not impact bacterial localization, nor did CTL targeting of APCs cause any decrease in viability of surface-attached bacteria (data not shown). Therefore, CTL killing of *Y. pseudotuberculosis*-associated APCs is insufficient to directly influence bacterial uptake or viability in this cell culture system.

### CTLs Indirectly Restrict *Y. pseudotuberculosis* by Marking APCs with Attached Bacteria for Phagocytic Removal

**Figure 8:**
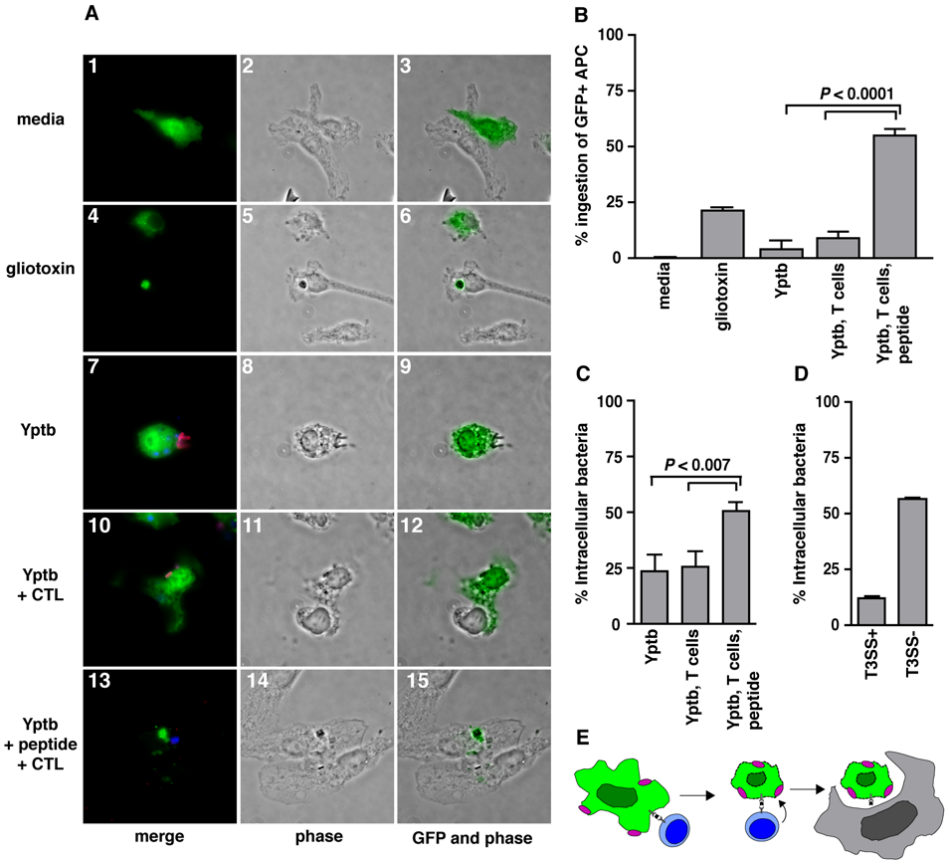
CTL Targeting Eliminates Both APCs and APC-Associated *Y. pseudotuberculosis* by Bystander Phagocytosis. (A) Immunofluorescence images of GFP^+^ APCs, pre-treated with media (panels 1–3), apoptosis-inducing gliotoxin (panels 4–6), *Y. pseudotuberculosis* (panels 7–9), *Y. pseudotuberculosis* and CrpA-specific CTLs (panels 10–12), or *Y. pseudotuberculosis*, CrpA_63–71_ peptide and CrpA-specific CTLs (panels 13–15), then exposed to IFNγ-activated macrophages. Panels from left to right show merged images of GFP^+^ APCs (green) with extracellular *Yersinia* (pink) and intracellular *Yersinia* (blue) (merge), phase contrast (phase), and phase contrast merged with GFP^+^ APCs (GFP and phase). Arrowheads indicate GFP^+^ cells, arrows indicate bacteria. White bar represents 10 µm. (B) Quantification of GFP^+^ host cells phagocytosed by activated macrophages. GFP^+^ cells were scored as unattached, attached or ingested by GFP^−^ activated macrophages and % ingested GFP^+^ host cells calculated. (C) Quantification of localization of *Y. pseudotuberculosis* associated with GFP^+^ APCs was performed similarly as described in Figure 7 legend: bacteria associated with GFP^+^ APCs were scored for intra- and extracellular localization and % intracellular bacteria calculated. Results shown are the mean and SEM of three triplicate samples per experiment; results are representative of three separate experiments. (D) Quantification of intracellular bacteria in macrophages exposed to TTSS^+^ or TTSS^−^ *Y. pseudotuberculosis* (no CTLs or peptide present). (E) Model of CTL restriction of *Y. pseudotuberculosis*: CTLs target bacteria-associated antigen-presenting cells for killing, apoptotic cells and attached bacteria are then engulfed and removed by neighboring phagocytes.

We next tested if CTLs could eliminate *Y. pseudotuberculosis* by targeting APCs and attached bacteria for phagocytosis by activated macrophages. We first tested whether recognition of dead cells by activated phagocytes could be readily demonstrated. Media-treated (viable) GFP^+^ cells resisted engulfment, whereas gliotoxin-treated (apoptotic) GFP^+^ cells were efficiently internalized by GFP^−^ IFNγ-activated macrophages (**Figure 8A**, panels 1–3 media, panels 4–6 gliotoxin; **Figure 8B**). Thus, this assay could be used to analyze the role of CTLs in promoting engulfment by activated phagocytes. To this end, GFP^+^ APCs with attached extracellular bacteria were exposed to CTLs, incubated with nonfluorescent activated macrophages, and uptake of apoptotic corpses and bacteria was quantified.

Internalization of bacteria and GFP^+^ APCs by the activated macrophages required CTL targeting of the APCs. In the absence of CTLs, *Y. pseudotuberculosis* exposure was insufficient to mark GFP^+^ APCs for engulfment by neighboring activated macrophages (**Figure 8A**, panels 7–9, **Figure 8B**). Similarly, APCs exposed to both bacteria and CTLs in the absence of peptide resisted engulfment (**Figure 8A**, panels 10–12), as only 9.2+1.8% of GFP^+^ APCs harboring bacteria were engulfed by activated macrophages if there was no peptide present (**Figure 8B**). However, GFP^+^ APCs harboring *Y. pseudotuberculosis* were phagocytosed at a high frequency, with 55.6±3.2% of cells internalized, if they were challenged with peptide and targeted by CTLs prior to exposure to the activated macrophages (**Figure 8A**, panels 13–15; **Figure 8B**). As CTL targeting enhanced engulfment of GFP^+^ APCs, we then determined if the bacteria associated with APCs were altered for localization, using the previously described antibody protection assay. CTL targeting of GFP^+^ APCs significantly enhanced bacterial internalization by activated macrophages (**Figure 8A**, panels 13–15, **Figure 8C**, third column), as compared to the absence of targeting (**Figure 8A**, panels 10–12; **Figure 8C**, second column). Phagocytosis of APC-associated *Y. pseudotuberculosis* after CTL targeting (**Figure 8C**, third column) was comparable to that observed with bacteria lacking the TTSS (**Figure 8D**, second column). Eliminating the possibility that bystander phagocytosis was limited to those APCs already harboring intracellular bacteria, the non-phagocytosed APCs displayed the same proportions of intracellular bacteria regardless of CTL targeting (**Figure S3**). Thus, CTL killing of *Y. pseudotuberculosis*-associated APCs defeats the anti-phagocytic activities of Yops, and allows bystander phagocytes to co-engulf bacteria associated with dead host cells.

## Discussion

We show here that CD8^+^ T cells and perforin are involved in protection against *Yersinia pseudotuberculosis*. We also present evidence that CD8^+^ T cells target antigen-presenting cells with attached bacteria for bystander phagocytosis. These findings are significant because CD8^+^ T cells and perforin targeting of APCs are thought to clear intracellular pathogens, while *Yersinia* species are generally considered to be extracellular [18]. *Yersinia* express proteins that facilitate tight binding to phagocytes, then inject anti-phagocytic Yops to maintain an extracellular, but attached, locale. We propose a model for how CTLs counteract bacteria in this niche: CD8^+^ T cells kill *Yersinia*-associated host cells, thus allowing apoptotic cells to be ingested by neighboring host cells and attached bacteria to be co-engulfed (**Figure 8D**). This uptake could be a death knell for the bacteria, as it essentially trumps the anti-phagocytic Yops.

The observation that CD8^+^ T cells were activated by attenuated *Y. pseudotuberculosis* bacteria (**Figure 2**) suggested that these host cells might protect against *Yersinia*. Indeed, mice devoid of CD8^+^ T cells due to the absence of β2-microglobulin (**Figure 3**) were more susceptible to colonization by the attenuated *ksgA^−^* mutant, showing increased bacterial burden in systemic organs regardless of inoculation route. Interestingly, the *B2m^−/−^* mice did not display increased bacterial colonization in the Peyer's patches. Given our prior observations that the bacterial populations responsible for seeding systemic organs are derived from those bacteria present in both the intestinal lumen and the Peyer's patches [43], the increased bacterial burden in the systemic organs of *B2m^−/−^* mice suggests that the bottleneck of lumen dissemination has been widened in these mice, such that bacterial populations present in systemic organs should be more representative of those in the lumen, rather than the Peyer's patches. With regards to β2-microglobulin-dependent immune responses, the enhanced susceptibility of *B2m^−/−^* mice to diverse pathogens such as *Trypanosoma cruzi*, *Listeria monocytogenes*, *Chlamydia trachomatis*, *Mycobacterium tuberculosis* and *Klebsiella pneumoniae* [66]–[70] has been used to demonstrate a requirement for MHC I-restricted T cells in protection against infection. However, at least for *M. tuberculosis* infection, the defect of *B2m^−/−^* mice may be due to iron overload or hemochromatosis [71], as β2-microglobulin associates with the MHC I family member HFE, which is involved in iron homeostasis [72]. Given that hemochromatosis is a risk factor for disseminated infection by enteric *Yersinia* in humans [54], the increased susceptibility of *B2m* ^−/−^ mice to oral inoculation with attenuated *Y. pseudotuberculosis* (**Figure 3**) may only partially result from the absence of CD8^+^ T cells. We did observe that knockout animals inoculated via a parental, i.e. non-oral, route also showed increased bacterial burden in systemic tissues, indicating that the enhanced susceptibility of the knockout mice cannot solely be explained by enhanced intestinal dissemination. It is also possible that the lack of natural killer T cells in *B2m^−/−^* mice [73] may contribute to the increased susceptibility to attenuated *Y. pseudotuberculosis* in this model. To more precisely examine the requirement for CD8^+^ T cells in protecting against *Y. pseudotuberculosis*, we tested additional mouse models of CD8 deficiency. The failure of CD8-depleted naïve mice to resist colonization with attenuated *Y. pseudotuberculosis* or CD8-depleted immune mice to survive virulent challenge (**Figure 4**) demonstrates that CD8^+^ cells are required for protective anti-*Y. pseudotuberculosis* immune responses. Similar observations have been made for *Y. pestis*, where CD8^+^ T cells help protect *Y. pestis*-immune mice from challenge [35],[36].

The mechanisms used by T cells to protect hosts against challenge with *Yersinia* are undefined, as tools to probe *Yersinia*-specific T cell responses are lacking. Yop proteins are obvious candidates for natural antigens recognized by T cells, by virtue of being directly translocated by the bacteria into the host cell cytoplasm, a feature that has been exploited to deliver Yop protein fusions into host cells to stimulate antigen-specific CD4^+^ and CD8^+^ T cells [74],[75]. Additionally, T cells specific to Yops have been isolated from *Y. pseudotuberculosis*-infected rats [76] or Yop-immunized mice [77]. This indicates that Yops can be processed into MHC I-binding peptides, and that virulence factors comprise a subset of natural antigens. Regardless of antigenic specificity, CTLs likely use multiple mechanisms to eliminate *Yersinia*. For instance, IFNγ and other CTL-secreted cytokines activate anti-microbial function in phagocytes, allowing them to ingest nearby bacteria [38]. T cell-secreted IFNγ does contribute to protection against *Y. pestis* [78] and *Y. enterocolitica* [34]. However, cytokine-mediated bystander action cannot be the sole explanation for restriction of *Y. pseudotuberculosis*, as cytolytic T cells (or other perforin-expressing killer cells) are clearly involved in protection (**Figure 5**). *PKO* mice are known to be defective for clearing intracellular pathogens such as lymphocytic choriomeningitis virus [79], *L. monocytogenes* ([80], *M. tuberculosis* [81] and *Leishmania amazonensis* [82] but our observation that perforin-dependent mechanisms limit *Y. pseudotuberculosis* is unique, given the bacteria's preferred extracellular locale.

We considered the possibility that CD8^+^ T cells and perforin function to restrict *Yersinia* in a conventional manner, that is, to eliminate host cells harboring intracellular bacteria. *Y. pseudotuberculosis* and *Y. pestis* have been shown to replicate inside cultured macrophages if the bacteria are deficient or repressed for Yop expression [59],[60]. The importance of intracellular replication for virulence in animal hosts is unclear, and has never been demonstrated. One expectation of this model is that the absence of CTLs results in an increased likelihood of bacteria being found in an intracellular locale. However, we failed to observe an increase in intracellular bacteria in the absence of CTLs or CTL function (**Figure 6B, C**), suggesting that CD8^+^ T cells are not functioning in a conventional manner to limit *Yersinia* replication. On the other hand, this does not eliminate the possibility that during the priming phase of immune responses, antigen-presenting cells harboring intracellular bacteria stimulate *Yersinia*-specific CD8^+^ T cell responses. As our experiments are directed toward asking how immunity is controlled by CD8^+^ cells at times significantly past the priming stage, we cannot rule out the possibility that intracellular bacteria are critical for triggering CD8^+^ T cell responses in a naïve animal.

In an immune animal, another alternative model posits that macrophages harboring intracellular bacteria are recognized and killed by T cells in a perforin-dependent fashion, and this leads to local inflammation that activates bystander phagocytes to clear extracellular bacteria. Arguing against this possibility is the observation that apoptotic cells are rapidly removed after *in vivo* CTL targeting [83] to prevent the pathogenic inflammation that results when apoptotic cells proceed to secondary necrosis [84]. Given these observations, and evidence showing that *Yersinia* localizes predominantly extracellularly (**Figure 6**) [24]–[26],[61], we favor a model whereby CTLs and perforin function to protect against extracellular *Yersinia*.

How would killing of host cells with attached extracellular *Y. pseudotuberculosis* lead to clearance of bacteria? At least two hypotheses exist. Firstly, perforin targeting of infected host cells may directly eliminate bacteria, either by killing bacteria on the host cell surface or by inducing uptake of attached bacteria. This model is consistent with observations that perforin insertion in host cells induces a wounded membrane response, in which lysosomes exocytose and release lysosomal contents on the cell surface [85]. The T3SS can also induce membrane wounding, which leads to increased uptake of *Y. pseudotuberculosis* lacking Yops [86]. Despite these observations, we found no evidence that CTL targeting of host cells with TTSS^+^ surface-bound *Y. pseudotuberculosis* altered the extracellular localization or viability of the bacteria, even at low CTL-to-host cell ratios or short incubation times to lower the effective dose of perforin (**Figure 7**, data not shown). A second hypothesis is that T cell-induced cytolysis of *Y. pseudotuberculosis*-associated host cells could indirectly eliminate the attached bacteria. In this model, cytolysis marks the target cell for removal by neighboring phagocytes, allowing cell-associated bacteria to be phagocytosed along with the dead cell. This outcome is similar to what is presumed to happen to the intracellular pathogen *L. monocytogenes* after CTL targeting. T cell cytolysis releases the intracellular bacteria from infected APCs, allowing bystander phagocytes to clear released bacteria and thus prevent the bacteria from spreading to infect nearby cells [87]. While we did not observe that CTL targeting caused *Y. pseudotuberculosis* to be released from cells (data not shown), we did observe that bacteria associated with CTL-targeted cells were engulfed by bystander phagocytes (**Figure 8A, C**) and presumably destroyed, although limitations of the cell culture model prevented this analysis. Thus, in this model, the propensity of *Y. pseudotuberculosis* to intimately associate with host cells also renders *Yersinia* susceptible to phagocytic removal after CTLs kill the bacteria-associated host cells (**Figure 8D**). Other extracellular pathogens are also restricted by CD8^+^ T cells. Uropathogenic *Escherichia coli* (UPEC), a causative agent of urinary tract infections (UTIs), binds tightly to bladder epithelial cells and lives in part extracellularly [88]. It was recently shown that in a mouse UTI model, UPEC colonization of the bladder stimulates CD8^+^ T cell responses, which contribute to clearance of the pathogen [89], albeit by an undescribed mechanism. It is tempting to speculate that the mechanism described here, in which extracellularly attached bacteria are phagocytosed as bystanders to APC elimination, may be a common strategy to restrict this class of pathogens.

In addition to CTL killing, it is likely that other mechanisms of host cell death restrict *Yersinia* replication. Interestingly, the YopJ/P protein expressed by *Yersinia* can induce apoptotic death in macrophages and dendritic cells [90]–[93]. YopJ/P either de-ubiquitinates or acetylates members of the mitogen-activated protein kinase (MAPK) and nuclear factor kappa B (NFκB) signaling pathways, resulting in suppression of inflammatory cytokine production and induction of cell death in infected phagocytes [94]. Although *Yersinia* lacking YopJ/P are minimally affected for virulence [95]–[98], recent work demonstrates that *Y. pseudotuberculosis* and *Y. pestis* strains that hypersecrete YopJ or express the more cytotoxic YopP variant cause more death in cultured cells and have lowered virulence in the mouse model [99],[100]. As enhanced apoptosis is thus restrictive for *Yersinia* replication, host cell death may represent an important aspect of the anti-*Yersinia* immune response. There is precedent for host cell death being protective against bacterial infection. *S. typhimurium* kills phagocytes by pyroptosis, a caspase-1-dependent death mechanism [101]. Caspase-1-deficient mice are more susceptible to *S. typhimurium* colonization than are wild-type mice [102], demonstrating that caspase-1-dependent death, or other caspase-1-dependent functions, are required to protect hosts from *Salmonella*. Furthermore, in a mouse model of *Streptococcus pneumoniae* lung colonization, inhibition of macrophage apoptosis was shown to increase bacterial burden in the lung and bloodstream, indicating that macrophage cell death mediates bacterial clearance [103]. Our work provides further evidence that eukaryotic cell death, in this case induced by host functions such as perforin, may serve to limit bacterial infection and reduce disease. The relative importance of the combined contributions of host- and pathogen-induced cell death upon *Yersinia* infection remains to be determined.

## Materials and Methods

All animal use procedures were in strict accordance with the NIH Guide for the Care and Use of Laboratory Animals and were approved by the Tufts University Institutional Animal Care and Use Committee.

### Mouse Strains and Procedures

B6.129-*B2m^tm1Jae^* (β2-microglobulin-deficient) and age-matched C57BL/6 mice were obtained from Taconic Farms (Germantown, NY), while C57BL/6-*Prf1^tm1Sdz^*/J (perforin-deficient), B6.129S2-*Cd8a^tm1Mak^*/J (*CD8* ^−/−^) mice and all other C57BL/6 mice were obtained from The Jackson Laboratory (Bar Harbor, ME). C57BL/6-Tg(CAG-EGFP)1Osb/J (transgenic EGFP^+^) mice were a kind gift from the laboratory of Dr. Diana Bianchi, Tufts University Medical Center. 8–10 week-old female mice were used for all experiments and were allowed to acclimatize for 5–7 days prior to use. Mice were housed in sterile specific-pathogen-free conditions. For depletion studies, mice were injected intraperitoneally with 200 µg monoclonal antibodies against murine CD8 in 200 µL phosphate-buffered saline (PBS), at days 3 and 1 prior to infection, then days 1, 4, 7, and 10 post-infection; control mice received PBS only [55]. Antibodies against murine CD8 (Ly-2.2, clone 2.43, American Type Culture Collection) were purified from hybridoma supernatants (Dr. Douglas Jefferson, Tufts University Medical Center) and determined to be endotoxin-free (data not shown). The CD8 deficiency status of the knockout or depleted mice was confirmed by fluorescence-activated cell sorting (FACS) analysis of spleen cells stained with mouse antibodies against CD8 (see Flow Cytometry below); depleted mice routinely displayed a 97–98% reduction in splenic CD8^+^ T cells, while knockout mice contained no detectable CD8^+^ cells (data not shown).

### Bacterial Strains

Two serotype III *Y. pseudotuberculosis* strain backgrounds were used: YPIII pIB1 [43] and IP2666 [60],[61]; YPIII pIB1 has a mutation in the *phoP* gene, while IP2666 encodes a functional allele [62]. YPIII pIB1^−^ and IP2666c, both of which lack the *Yersinia* virulence plasmid, have been described previously [61]. The *ksgA^−^* strain, which harbors a transposon in the kasugamycin resistance gene [40] and the *dam^OP^* strain, which harbors a kanamycin resistance gene in the *dam* gene and a plasmid constitutively overexpressing *Escherchia coli* Dam methylase [48] were derived from YPIII pIB1. The *dam^OP^* strain was a kind gift from Dr. Michael Mahan, University of California Santa Barbara, and *Salmonella enterica* serovar Typhimurium strain SL1344 was kindly provided by Dr. Brad Cookson, University of Washington.

### *In Vivo* Inoculations, Survival and Organ CFU Assays

For mouse inoculations, *Y. pseudotuberculosis* strains were grown overnight in L broth at 26°C with aeration, pelleted and resuspended in PBS to the desired concentration. Mice were deprived of food for 18–20 hours and inoculated by oral gavage (feeding needle no. 7920; Popper & Sons, Inc., New Hyde Park, NY), first with 100 µL 5% sterile sodium bicarbonate, to buffer stomach contents, then with 200 µL of 5×10^8^ colony-forming units (CFU) of *ksgA^−^* or YPIII pIB1 *dam^OP^* for immunization studies, or 5×10^9^ CFU of virulent YPIII pIB1 for challenge experiments. Alternatively, mice were inoculated intravenously via the lateral tail vein (30½ gauge needle, Becton Dickinson & Co., Franklin Lakes NJ), with 200 µL of 10^2^ CFU *ksgA^−^* or 10^3^–10^4^ CFU virulent YPIII pIB1, IP266 or 10^2^ SL1344. For survival experiments, animals were sacrificed upon displaying signs of morbidity (hunched, scruffy fur, lethargy) and scored as non-surviving. To determine CFU levels in organs, mice were sacrificed by cervical dislocation at the indicated time points, tissues harvested, placed in pre-weighed tubes containing sterile PBS, and weighed to determine tissue weight. Tissues were mechanically homogenized using a tissue homogenizer (Omni, Marietta GA) and 100 µL of dilutions of tissue homogenate were plated on L agar plates containing 1 µg/mL of irgasan [104]. After 48 hours incubation at room temperature, CFU were enumerated and normalized to the gram weight of each tissue. As each animal may have an organ weight different from the other animals, the CFU values at or below the limit of detection (10 CFU per mL of organ homogenate) can be different between individual animals.

### Enzyme-Linked ImmunoSorbent Assays (ELISAs)

To generate a *Y. pseudotuberculosis* total antigen preparation for indirect sandwich ELISA [55], YPIII pIB1 bacteria were grown with aeration in either high calcium medium at 26°C (Luria broth, 18–20 hours at 26°C) to inhibit Yop production or low calcium medium at 37°C (2× YT broth with 20 mM sodium oxalate and 20 mM MgCl_2_, 1.5 hours at 26°C, 1.5 hours at 37°C) to induce Yop production, then pelleted, resuspended in PBS and sonicated on ice (Sonic Dismembrator, Fisher Scientific, Waltham MA) every other 30 seconds for 5 minutes. Suspensions were cleared of unbroken cells by low-speed centrifugation (Eppendorf, Westbury NY), and concentration determined by bicinchoninic acid assay (Pierce, Rockford IL). Samples prepared from bacteria grown in high or low calcium conditions were pooled to constitute a total *Y. pseudotuberculosis* antigen preparation and used to coat 96 well flat-bottom plates (Corning Costar, Lowell MA) at a concentration of 10 µg/mL. Standards for isotype ELISAs included purified mouse IgA, IgG1 or IgG2a (BD Biosciences Pharmingen), which were coated on plates at 1 µg/mL. After overnight incubation of antigen and standards at 4°C, plates were blocked with 10% fetal calf serum, and mouse serum samples were applied in serial dilution. Sera were prepared by centrifugation (Microtainer Serum Separator Tube, Becton Dickinson & Co., Franklin Lakes NJ) of mouse blood obtained by tail vein incision or cardiac puncture. Bound antibodies were probed with alkaline phosphatase (AP)-conjugated goat antisera against “total” (IgD+IgM+IgG+IgA) and IgA, IgG1, and IgG2a isotype mouse antibodies (SouthernBiotech, Birmingham AL), and bound AP detected with BluePhos substrate solution (KPL, Gaithersburg MD) at an optical density (OD) of 595 nm.

### Flow Cytometry

Single cell suspensions of Peyer's patches and mesenteric lymph nodes were prepared by gently dissociating the tissue through 70 µm nylon mesh (Falcon, BD Biosciences Discovery Labware, San Jose CA). After removal of red blood cells by PharmLyse, approximately 10^6^ cells were stained in the presence of Fc block (clone 2.4G2) with antibodies to murine CD4 (L3T4, clone RM4-4, FITC conjugated), CD8 (Ly-2, clone 53-6.7, PE conjugated) and CD69 (Very Early Activation Antigen, clone H1.2F3, PE-Cy7 conjugated). Stained cells were fixed with 2% paraformaldehyde (Electron Microscopy Services, Hatfield PA), and data acquired on a FACSCalibur (BD Biosciences Immunocytometry Systems, San Diego CA). All flow cytometry reagents and antibodies were purchased from BD Biosciences Pharmingen (San Jose CA).

### *Ex Vivo* Gentamicin Protection Assays

Single cell suspensions of spleens from animals exposed to bacteria were prepared by gently dissociating the tissue through 70 µm nylon mesh (Falcon, BD Biosciences Discovery Labware, San Jose CA). The samples were assayed for gentamicin-protected bacteria using two similar protocols. The first protocol was the same as previously described [61]: samples were divided in two 0.5-ml aliquots: one aliquot was treated with 100 µg/ml gentamicin while the other aliquot was left untreated. After 2 hour at 37°C in 5% CO_2_, the cells were washed three times with PBS, lysed with 100 µl of 1% Triton-X-100 for 5 min followed by the addition of 900 µl PBS, then lysates diluted and plated for colon-forming units to determine the number of total and gentamicin-protected bacteria. The second protocol is similar to the first protocol, except aliquots for CFU determination were removed from the same sample prior to and then after 1 hour gentamicin treatment. The two protocols yielded comparable results with regards to levels of % gentamicin-protected bacteria (value equal to the number of gentamicin-protected CFU divided by the total number of CFU multiplied by 100).

### Mammalian Cell Culture

Primary cultures of bone marrow-derived macrophages were established from femurs and tibias of C57BL/6 and EGFP^+^ mice. Bone marrow cells were grown for 6 days in high-glucose DME medium, supplemented with 20% heat-inactivated fetal calf serum, 10% NIH 3T3-CSF or 30% L-cell conditioned cell supernatant, 2 mM L-glutamine, 1 mM sodium pyruvate, and 55 µM β-mercaptoethanol (Invitrogen, Carlsbad CA). Macrophages were cultured in 60×15 mm or 150×25 mm petri dishes (Nunc LabTek Fisher Scientific, Rochester NY) prior to reseeding in tissue culture dishes for individual assays (below). Where indicated, macrophages were activated with 1 ng/mL recombinant mouse IFNγ (R&D Systems, Minneapolis MN) for 24 hours or treated with 5 µM gliotoxin (Calbiochem, San Diego CA) for 4–6 hours. The CrpA_63–71_-specific T cell line was derived from *Chlamydia trachomatis*-immune C57BL/6 mice (W.P. Loomis and M. N. Starnbach, unpublished) and was maintained in RPMI 1640 medium supplemented with 10% heat-inactivated fetal calf serum (Hyclone, Logan UT), 5% rat T-STIM with ConA (IL-2 supplement, BD Biosciences Discovery Labware, San Jose, CA), 50 mM methyl-α-D-mannopyranoside (Calbiochem, San Diego, CA), 2 mM L-glutamine, 50 µM β-mercaptoethanol, 100 u/mL penicillin and 100 µg/mL streptomycin (Invitrogen, Carlsbad CA). Every 7 days, T cells were restimulated with irradiated syngeneic splenocytes and irradiated EL4 thymoma cells stably transfected with *crpA*. Non-transfected EL4 thymoma cells, used as control antigen-presenting cells, were maintained in RPMI 1640 media supplemented exactly as for CrpA-specific CD8^+^ T cells except for the absence of IL2 supplement; 600 µg/mL geneticin (Invitrogen, Carlsbad CA) was used for selection of EL4-*crpA* transfectants.

### Gentamicin Protection Assays with Bone-Marrow Derived Macrophages

The protocol is similar to that described previously [60]. C57BL/6 bone-marrow derived macrophages (day 6 post-harvest/derivation) were seeded onto glass coverslips (Fisherbrand, Fisher Scientific, Waltham MA) in 24 well plates at 2×10^5^ per well and allowed to adhere overnight. Bacteria were grown with aeration in Luria broth for 18–20 hours at 26°C, and PBS-washed samples added to macrophage-containing wells at an estimated multiplicity of infection of 25∶1. Bacteria were spun onto macrophages at 1000 rpm, then co-cultures incubated at 37°C/5% CO2 for 40 minutes. After washing twice in PBS, cells were overlaid with media containing 100 µg/mL gentamicin (t = 0) and incubated for 1 hour. After washing twice in PBS, cells were overlaid with media containing 5 µg/mL gentamicin (t = 1) and incubated further. At the indicated timepoints, cells were washed twice with PBS and lysed with 0.2 mL 1% Triton X-100. Following the addition of 0.8 mL PBS, samples were serially diluted and plated to determine CFU. The % gentamicin bacteria results from the number of gentamicin-protected CFU divided by the total number of CFU multiplied by 100.

### Cytotoxicity Assays

Bone-marrow derived macrophages were seeded to 96 well plates at 10^4^ cells per well, allowed to adhere overnight, pulsed with or without 100 nM CrpA_63–71_ peptide (Biosynthesis Incorporated, Lewisville TX) for 1 hour, and then excess peptide was washed away. The thymoma EL4 cells were pulsed with peptide in suspension, then seeded similarly as macrophages. CrpA_63–71_-specific CD8^+^ T cells were harvested at five days post-restimulation and added to antigen-presenting cells at the indicated effector∶target cell ratios, co-cultured for 4 hours, after which the plates were centrifuged, supernatant removed and assayed for the presence of the cytoplasmic enzyme lactate dehydrogenase using the Cytotox 96 assay kit (Promega, Madison WI) according to the manufacturers' recommendations. % Cytotoxicity was calculated as follows: 100×[(experimental release – effector T cell spontaneous release – target cell spontaneous release)/(target cell maximum release – target cell spontaneous release)].

### CTL Targeting of *Yersinia*-associated APCs in Culture

CTL targeting used two experimental procedures. In the first protocol, C57BL/6 bone-marrow derived macrophages were seeded onto glass coverslips (Fisherbrand, Fisher Scientific, Waltham MA) in 24 well plates at 10^5^ per well, allowed to adhere overnight, and then incubated with media containing *Y. pseudotuberculosis* (grown in low-calcium medium to induce Yop expression, diluted to give a multiplicity of infection of 10∶1) and 100 nM CrpA_63–71_ peptide. After 1 hour, macrophages were washed three times with PBS to remove unbound bacteria and peptide and CrpA_63–71_-specific CD8^+^ T cells (harvested at five days post-restimulation) added at an effector∶target cell ratio of 2∶1. After 15 minutes, the cells were fixed with 4% paraformaldehyde and processed for immunofluorescence microscopy (below). In the second protocol, GFP^+^ bone marrow derived macrophages, plated in 60×15 mm Petri dishes at approximately 5×10^6^ cells per dish, were challenged with *Y. pseudotuberculosis* at an MOI of 10∶1 and pulsed with 100 nM CrpA_63–71_ peptide for 1 hour, then washed three times with PBS. Flow cytometry-sorted CrpA-specific CTLs were added to the infected antigen-presenting cells at an effector∶target cell ratio of 2∶1 and co-cultures incubated for 1 hour. All cells were then harvested and processed for phagocytosis assays (below). Viable CTLs were sorted away from dead irradiated feeder cells based on forward scatter and side scatter differences (Tufts University Department of Pathology Flow Cytometry Core) before being added to infected antigen-presenting cells; sorted CTLs were as effective as unsorted CTLs at targeting antigen-presenting cells for LDH release (data not shown).

### Phagocytosis Assay

C57BL/6 bone-marrow derived macrophages were seeded onto glass coverslips (Fisherbrand, Fisher Scientific, Waltham MA) in 24 well plates at 0.5×10^5^ per well in medium containing 1 ng/mL IFNγ and allowed to adhere overnight. Substrate cells were then added to IFNγ-activated macrophages at a ratio of 1∶1 substrate cell∶activated phagocyte and incubated together for 1–1.5 hours, after which time the cells were fixed with 4% paraformaldehyde and processed for immunofluorescence microscopy (below). Coverslips with fixed stained cells were mounted with Fluoroguard Antifade Reagent (Bio-Rad, Hercules, CA) and individual cells inspected using a Plan-NeoFluar 100×/1.3 Ph3 objective on a Zeiss Axioskop microscope (Carl Zeiss, Thornwood, NY) or using a Plan-Fluor 100×/1.3 Ph3 objective on a Nikon Eclipse TE300 inverted microscope (Nikon, Tokyo, Japan). Images were captured using a Hamamatsu Orca II camera (Hamamatsu Photonics, Hamamatsu City, Japan). For each condition, 3 coverslips and 100 GFP^+^ substrate cells per coverslip were inspected and GFP^+^ cells scored for ingestion by non-GFP IFNγ-activated macrophages. % ingestion was calculated as 100×(ingested GFP^+^ cells/total GFP^+^ cells).

### Immunofluorescence Protection Assays of Bacterial Uptake or Intracellular Replication

Immunofluorescence-based bacterial uptake and replication assays were performed similarly to those described previously [105],[106]. After fixation, cells were blocked with 4% goat serum and probed with rabbit polyclonal antibodies against *Y. pseudotuberculosis* serogroup O3 followed by goat anti-rabbit IgG conjugated to Alexa 594 (for uptake assays) or Cascade Blue (for intracellular replication assays) (Invitrogen Molecular Probes, Eugene OR). The cells were then permeabilized by immersion in ice-cold methanol for 10 seconds, blocked once more and reprobed with anti-*Y. pseudotuberculosis* antibodies followed by goat anti-rabbit IgG conjugated to Cascade Blue (for uptake assays) or FITC (for intracellular replication assays) (Invitrogen Molecular Probes, Eugene OR). Where indicated, for uptake assays, macrophages were stained with rat antibodies against murine CD11b (Integrin α_M_ chain, clone M1/70, BD Biosciences Pharmingen, San Jose CA) followed by goat anti-rat IgG conjugated to FITC (Zymed, San Francisco CA). For both uptake and intracellular replication assays, 3 coverslips and 100 infected host cells per coverslip were inspected by fluorescence microscopy as described above. To determine uptake, cell-associated bacteria scored for localization and % intracellular bacteria was calculated as 100×(intracellular bacteria/total bacteria). To determine intracellular replication, macrophages were scored for the number of intracellular bacteria per phagosome, and the % replicative phagosomes calculated as 100×(phagosomes with 10+ bacteria/total bacteria-containing phagosomes).

### Data and Statistical Analysis

FlowJo (Tree Star, Ashland OR) was used to analyze flow cytometry data. Prism (GraphPad Software, La Jolla CA) was used for graphing and statistical analysis. To calculate the half-maximal antibody titer of total anti-*Yersinia* antibodies, OD_595_ and log-transformed serial dilution values were fit to a sigmoidal dose-response curve. Survival curves were estimated using the Kaplan Meier method and significance calculated using the log-rank test. The nonparametric Mann-Whitney U test and unpaired Student's t test were used to determine statistical differences between groups of data from animal and tissue culture experiments, respectively.

## Supporting Information

Figure S1 *Y. pseudotuberculosis ksgA^−^* is Attenuated for Systemic Organ Colonization Following Intravenous Delivery. C57BL/6 mice were intravenously inoculated with 2×10^2^ CFU of YPIII pIB1 (virulent parental strain), *yopB^−^* and *ksgA^−^* bacteria and mice sacrificed at day 5 post-inoculation. The number of bacteria in the spleen (A) and liver (B) was determined by plate assay and normalized to gram tissue weight. Each symbol indicates one mouse and the bars indicate median values.
(0.51 MB PDF)

Figure S2 *Y. pseudotuberculosis* strain YPIII pIB1 fails to survive or replicate inside bone-marrow-derived macrophages. C57BL/6 macrophages were challenged with the indicated strains for 1 hour, washed and treated with gentamicin, then the number of (A) gentamicin-protected bacteria enumerated at the indicated times post-challenge or (B) replicative phagosomes (% of phagosomes containing 10+ bacteria as determined by immunofluorescence microscopy) were enumerated at 24 hours post-challenge. See Materials and Methods for experimental details. Values shown represent the average of triplicate samples in a given experiment, error bars indicate the SEM, error bars are not visible in the growth curve results to their small size. Results are representative of 3 experiments.
(0.51 MB PDF)

Figure S3 The population of CTL-targeted GFP^+^ APCs excluded from bystander phagocytosis possesses equivalent levels of intracellular bacteria relative to non-targeted populations. Quantification of localization of *Y. pseudotuberculosis* associated with GFP^+^ APCs was performed similarly as described in Figure 7 legend: bacteria associated with all GFP^+^ APCs (A, duplicate of Figure 8C for comparison) or non-engulfed GFP^+^ APCs (B) were scored for intra- and extracellular localization and the % intracellular bacteria calculated.
(0.50 MB PDF)
