# Supplementary figures and images for: CD8+ T Cells Restrict Yersinia pseudotuberculosis Infection: Bypass of Anti-Phagocytosis by Targeting Antigen-Presenting Cells
Source: PLoS Pathog. 2009 Sep 4;5(9):e1000573. doi: 10.1371/journal.ppat.1000573 (PMC2731216; doi:10.1371/journal.ppat.1000573)

Supplementary Figure 1

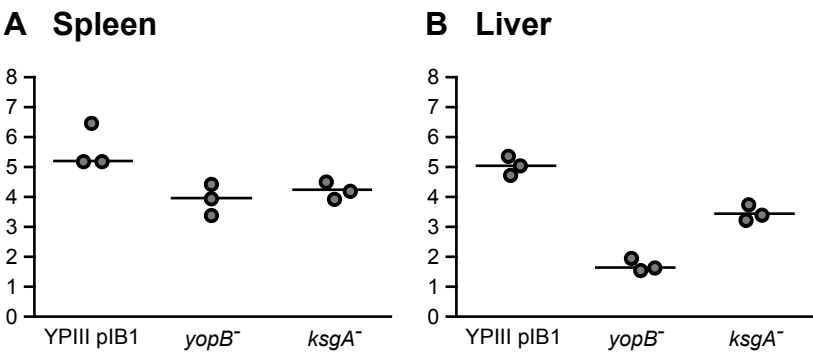

Supplement: Figure S1 — Y. pseudotuberculosis ksgA− is Attenuated for Systemic Organ Colonization Following Intravenous Delivery. C57BL/6 mice were intravenously inoculated with 2×10^2^ CFU of YPIII pIB1 (virulent parental strain), yopB− and ksgA− bacteria and mice sacrificed at day 5 post-inoculation. The number of bacteria in the spleen (A) and liver (B) was determined by plate assay and normalized to gram tissue weight. Each symbol indicates one mouse and the bars indicate median values. (0.51 MB PDF) [file ppat.1000573.s001.pdf]

## Supplementary Figure 2

**A**

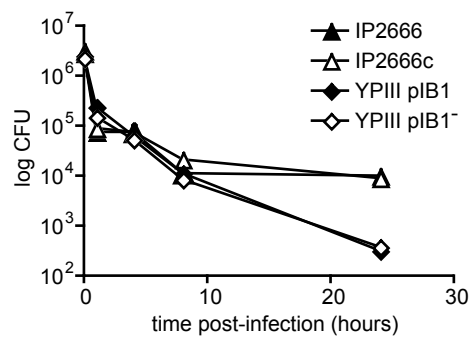

**B**

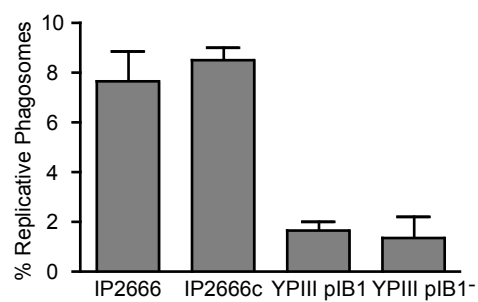

Supplement: Figure S2 — Y. pseudotuberculosis strain YPIII pIB1 fails to survive or replicate inside bone-marrow-derived macrophages. C57BL/6 macrophages were challenged with the indicated strains for 1 hour, washed and treated with gentamicin, then the number of (A) gentamicin-protected bacteria enumerated at the indicated times post-challenge or (B) replicative phagosomes (% of phagosomes containing 10+ bacteria as determined by immunofluorescence microscopy) were enumerated at 24 hours post-challenge. See Materials and Methods for experimental details. Values shown represent the average of triplicate samples in a given experiment, error bars indicate the SEM, error bars are not visible in the growth curve results to their small size. Results are representative of 3 experiments. (0.51 MB PDF) [file ppat.1000573.s002.pdf]

Supplementary Figure 3

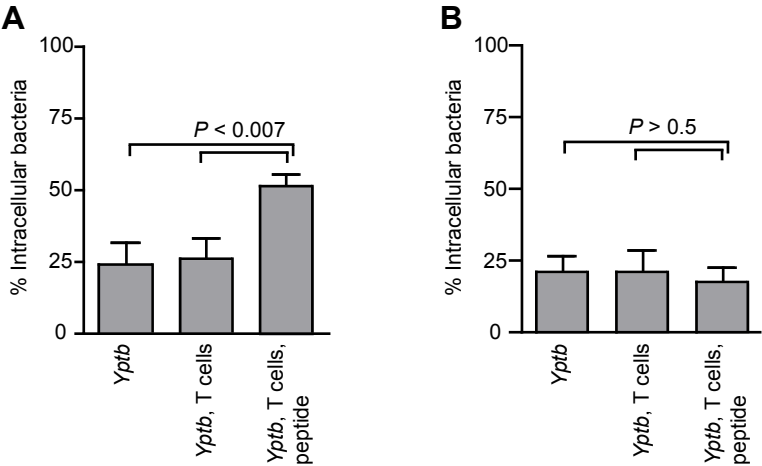

Supplement: Figure S3 — The population of CTL-targeted GFP+ APCs excluded from bystander phagocytosis possesses equivalent levels of intracellular bacteria relative to non-targeted populations. Quantification of localization of Y. pseudotuberculosis associated with GFP+ APCs was performed similarly as described in Figure 7 legend: bacteria associated with all GFP+ APCs (A, duplicate of Figure 8C for comparison) or non-engulfed GFP+ APCs (B) were scored for intra- and extracellular localization and the % intracellular bacteria calculated. (0.50 MB PDF) [file ppat.1000573.s003.pdf]
